# Applications of non-invasive brain stimulation in the treatment of children with cerebral palsy: a narrative review

**DOI:** 10.3389/fped.2026.1867739

**Published:** 2026-10-27

**Authors:** Ce Wang, Yuqiu Jia, Xiaoyan Ding, Dandan Wang, Qing Shi, Shasha Zhan, Zhicheng Bao, Lei Huang

**Affiliations:** 1 Department of Pediatrics, Kunshan NO.4 People’s Hospital, Kunshan, China; 2 Department of Rehabilitation Medicine, The Affiliated Taizhou Hospital of Nanjing Medical University, Taizhou First People’s Hospital, Taizhou, China; 3 Department of Rehabilitation Medicine, Kunshan NO.4 People’s Hospital, Kunshan, China; 4 Department of Rehabilitation Medicine, The Affiliated Kunshan Hospital of Jiangsu University, Kunshan First People’s Hospital, Kunshan, China

**Keywords:** cerebral palsy, children, noninvasive brain stimulation, repetitive transcranial magnetic stimulation, transcranial direct current stimulation

## Abstract

Noninvasive brain stimulation has emerged as a promising adjunctive strategy for pediatric neurorehabilitation, particularly for children with cerebral palsy. This narrative review, informed by a structured literature search, summarizes current evidence on transcranial direct current stimulation and repetitive transcranial magnetic stimulation in children with cerebral palsy, with emphasis on neurophysiological mechanisms, brain network targets, clinical outcomes, safety, and future translational directions. Cerebral palsy is a heterogeneous neurodevelopmental condition characterized by persistent motor impairment and frequent sensory, cognitive, communicative, behavioral, and musculoskeletal comorbidities. Although multidisciplinary rehabilitation remains the foundation of clinical management, improvements in higher level motor control, cognition, communication, and real world participation are often constrained by training intensity, adherence, developmental heterogeneity, and resource availability. Neuroimaging and electrophysiological studies indicate that children with cerebral palsy may present with corticospinal tract impairment, altered cerebellar thalamic cortical circuitry, interhemispheric inhibitory imbalance, and atypical development of frontoparietal executive control and language related networks. These findings provide a rationale for targeting the primary motor cortex, dorsolateral prefrontal cortex, and selected language related cortices with neuromodulatory interventions. At the mechanistic level, transcranial direct current stimulation and repetitive transcranial magnetic stimulation may influence cortical excitability, NMDA receptor mediated long term potentiation and long term depression like plasticity, interhemispheric inhibition, and functional connectivity within distributed neural networks. Current evidence from small to moderate sized randomized trials and recent systematic reviews suggests that these techniques, when combined with task specific rehabilitation, may provide additional benefits for gross motor function, gait, balance, upper limb dexterity, spasticity, and selected cognitive, dual task, and communication outcomes. Available data also suggest acceptable short term safety and tolerability when appropriate pediatric screening and recommended stimulation parameters are applied. This review summarizes the current research progress on noninvasive brain stimulation in children with cerebral palsy and highlights its potential value for optimizing individualized neurorehabilitation strategies and improving motor, cognitive, and communication related outcomes.

## 1 Introduction

Cerebral palsy (CP) is a group of neurodevelopmental disorders originating in the fetal or infant period that result from non-progressive disturbances or lesions in the developing brain. Its hallmark features are persistent disorders of movement and posture causing activity limitation (1). International consensus further emphasizes that CP is not merely a motor disorder; multisystem involvement, including sensory, cognitive, and communication impairments, epilepsy, and secondary musculoskeletal complications, is common in clinical practice, making CP a highly heterogeneous syndrome that spans the entire life course (2, 3). Epidemiological data indicate that the prevalence of CP among live births is approximately 1.5 to 3.0 per 1000 in most countries. A substantial proportion of affected children, in addition to limb motor limitations, also present with visual and auditory impairments, epilepsy, and cognitive and language difficulties. These comorbidities are closely associated with functional independence, academic performance, and social participation, yet may still be underestimated in routine follow up and rehabilitation decision making (4–6).

Current management of CP is centered on multidisciplinary comprehensive rehabilitation, including physical therapy, occupational therapy, speech and language therapy, orthotic management, and pharmacological interventions. These approaches have moderate level evidence for improving gross motor function, muscle tone, and certain activities of daily living (7, 8). However, several systematic reviews have shown that most rehabilitation therapies require long term, high frequency training to achieve clinically meaningful improvements. Treatment effectiveness is highly dependent on family resources and adherence, and traditional rehabilitation often reaches a plateau for complex functional domains such as higher order motor control, cognition, and communication, with limited additional gains thereafter (8, 9). Therefore, integrating adjunctive interventions that can directly modulate central nervous system plasticity into existing rehabilitation frameworks, so as to enhance training efficiency and raise functional ceilings, has become an important research and clinical priority.

Non-invasive brain stimulation (NIBS) comprises a rapidly advancing class of neuromodulation techniques, primarily including transcranial direct current stimulation (tDCS) and repetitive transcranial magnetic stimulation (rTMS). tDCS delivers low intensity direct current through scalp electrodes to modulate cortical excitability and plasticity in a polarity dependent manner. rTMS, based on electromagnetic induction, applies repetitive stimulation at specific frequencies and patterns to induce synaptic plasticity changes resembling long term potentiation or long term depression (10, 11). In adults, NIBS has demonstrated moderate to good efficacy as an adjunct to medication and rehabilitation training for post stroke motor and language impairments, Parkinson's disease, and depressive disorders, and it is generally safe and well tolerated when applied within recommended parameter ranges (12, 13).

The pediatric brain is characterized by heightened plasticity, which theoretically provides an advantage for NIBS in promoting neural network reorganization. At the same time, it raises greater concerns regarding dose response relationships, developmental stage specific mechanisms, and long term safety (14, 15). Existing systematic reviews suggest that tDCS and rTMS are overall safe and feasible in CP and other pediatric motor disorders following brain injury, with promising signals for outcomes such as upper limb function, gait, and balance. Nevertheless, most studies have focused primarily on gross motor outcomes, and evidence remains limited for more complex domains, including cognition, language, and cognitive motor dual task performance. In addition, stimulation parameters and target selection lack standardized guidelines (16, 17). Against this background, this narrative review aims to summarize the applications of NIBS, with an emphasis on tDCS and rTMS, in the treatment of children with CP. We outline the main techniques and their characteristics in neurological disorders, review brain network alterations in CP and the neurophysiological mechanisms of tDCS and rTMS, synthesize evidence on motor, cognitive, and language outcomes as well as safety, and discuss future research directions and translational strategies to provide clearer theoretical and evidence based support for target selection and individualized intervention protocols.

## 2 Methods

This review addresses the neurophysiological mechanisms of non invasive brain stimulation (NIBS) and their network level interpretations, together with clinical outcomes across motor, cognitive, language and communication, and dual task domains in children with cerebral palsy.

English language literature was searched in PubMed, Embase, Web of Science, and Google Scholar, and reference lists of included articles were screened to identify additional relevant studies. The search covered database inception to 25 December 2025. Search terms were grouped into condition and population, intervention, and outcomes or mechanisms, and combined using Boolean operators. An example PubMed search string was: (“cerebral palsy” OR “perinatal stroke” OR “hemiplegia”) AND (“child” OR “pediatric” OR “adolescent”) AND (“transcranial direct current stimulation” OR “tDCS” OR “repetitive transcranial magnetic stimulation” OR “rTMS” OR “TMS” OR “non invasive brain stimulation”).

Eligible studies included interventional research evaluating tDCS or rTMS in children and adolescents aged 0 to 18 years with cerebral palsy or closely related motor disorders attributed to non progressive pediatric brain injury. Eligible study designs included randomized controlled trials, controlled clinical studies, crossover studies, and prospective or retrospective studies reporting clinical outcomes after stimulation. Relevant systematic reviews and meta analyses were also included to support synthesis across outcomes and protocols. Studies were required to report at least one outcome in motor function, gait, balance, upper limb performance, spasticity or muscle tone, cognition, executive function, attention, language or communication, dual task performance, and or safety and tolerability outcomes. Exclusion criteria included non English publications, conference abstracts without sufficient methodological detail, case reports with fewer than five participants, and studies not involving tDCS or rTMS or not including an eligible pediatric population.

Records were screened at the title and abstract level, followed by full text assessment for eligibility. Two reviewers independently screened records and assessed full texts, disagreements were resolved by discussion, and a third reviewer adjudicated when consensus could not be reached. For studies meeting inclusion criteria, information extracted for narrative synthesis included participant characteristics and functional severity when reported, stimulation modality, target and stimulation parameters, number of sessions and total dose, concurrent training content, outcome measures and assessment time points, follow up duration, and adverse events. Adult studies and studies in other pediatric neurodevelopmental disorders were cited only when pediatric cerebral palsy evidence was limited, and were used as supplementary support for mechanistic interpretation and safety considerations.

## 3 Brain network alterations in children with cerebral palsy and targets of nibs

### 3.1 Multisystem characteristics of structural and functional network impairments

The pathological substrates of cerebral palsy include cortical and subcortical gray matter injury, perinatal white matter injury including periventricular leukomalacia, periventricular white matter lesions, and developmental brain malformations. These abnormalities may involve multiple systems, including the corticospinal tract, cerebellum, brainstem, cortico cortical circuits, and frontoparietotemporal connectivity (18, 19). Diffusion tensor imaging (DTI) studies indicate that children with spastic CP commonly exhibit reduced fractional anisotropy in the bilateral or unilateral corticospinal tract, reflecting compromised tract integrity. The severity of these alterations is closely associated with abnormal muscle tone, co contraction, and deficits in gross motor function (20). In this context, effective descending drive from the primary M1 to anterior horn motor neurons is weakened, while activity in compensatory pathways, such as the reticulospinal tract, may be upregulated. This pattern is considered an important neurobiological basis for motor phenotypes characterized by the coexistence of spasticity and weakness (21, 22).

Beyond descending motor pathways, the cerebellum and basal ganglia may also be affected, particularly in children with ataxic CP and in a subset of those with spastic CP. In these cases, reduced cerebellar volume and abnormal diffusion metrics in the cerebellar peduncles and cerebello thalamo cortical pathways are more prominent, and clinically they are often manifested as impaired postural control, increased gait variability, and reduced balance capacity (23). Functional MRI studies further suggest that, during lower limb motor tasks, children with CP show decreased functional connectivity within cerebello motor cortical networks. During relatively simple voluntary movement tasks, more widespread and non specific activation may be observed, suggesting that basic motor performance may require recruitment of additional neural resources but with lower network efficiency (24, 25). Collectively, M1 and cerebello cortical circuits can be considered key network nodes underlying motor impairment in CP and represent major potential targets for NIBS to modulate motor function.

### 3.2 Interhemispheric inhibitory imbalance and plasticity of motor networks

In hemiplegic cerebral palsy, imbalance in transcallosal interactions is thought to contribute to maladaptive reorganization of motor networks. Transcranial magnetic stimulation studies have shown increased transcallosal inhibition from the contralesional M1 to the ipsilesional M1, whereas cortical excitability and corticospinal output from the ipsilesional M1 are relatively reduced. This asymmetric inhibition is associated with poorer motor function of the affected limb (26). A similar pattern has been observed in children with hemiplegia secondary to perinatal ischemic stroke, in whom some individuals exhibit atypical motor representations, with increased bilateral limb output from the contralesional hemisphere, which may further diminish the contribution of the injured hemisphere (27).

Based on this model, NIBS in cerebral palsy commonly adopts two complementary strategies. One strategy is to enhance excitability and descending output of the ipsilesional M1 using anodal tDCS or high frequency repetitive transcranial magnetic stimulation. The other strategy is to suppress excessive activity of the contralesional M1 using cathodal tDCS or low frequency repetitive transcranial magnetic stimulation, thereby reducing pathological inhibition of the ipsilesional hemisphere and promoting restoration of interhemispheric functional balance (28, 29). Notably, the interhemispheric inhibition model may vary across etiologies and developmental stages, indicating substantial heterogeneity. Therefore, clinical application requires more refined stratification and individualized selection informed by neuroimaging and electrophysiological phenotypes.

### 3.3 Developmental alterations in cognitive and language networks

Beyond the motor system, children with cerebral palsy commonly exhibit cognitive and language impairments of varying severity. The neurobiological basis of these deficits primarily involves atypical development of prefrontal and parietal networks, as well as language related networks (30). Structural imaging studies suggest that a subset of children with cerebral palsy show reduced volume and compromised integrity of frontal white matter and the anterior portion of the corpus callosum, which are closely associated with poorer executive function, reduced attentional control, and slower processing speed (31, 32). Task based functional MRI and functional near infrared spectroscopy studies have shown abnormal activation patterns of the dorsolateral prefrontal cortex (DLPFC) and frontoparietal networks during working memory, inhibitory control, and dual task walking. These patterns are characterized by more widespread or higher magnitude activation compared with typically developing peers, together with reduced efficiency metrics associated with task performance, such as decreased task related desynchronization amplitude. This suggests that children with cerebral palsy may recruit greater neural resources to sustain relatively lower behavioral performance (33). Resting state studies have also reported altered functional connectivity between the default mode network and the frontoparietal control network, which may be related to attentional fluctuations and cognitive fatigue (34).

With respect to language, a substantial proportion of children with spastic or mixed cerebral palsy present with expressive language delay, dysarthria, and childhood apraxia of speech. Neuroimaging studies indicate structural and functional connectivity alterations involving the left inferior frontal gyrus (Broca area), the supramarginal gyrus, and their connections with the auditory cortex (35, 36). In some children, language lateralization is incomplete or atypical, with greater involvement of the right hemisphere in language processing. This pattern may reflect post injury redistribution of language function in early development, but it has also been associated with constraints in language efficiency and fine grained expressive abilities (37). Collectively, these findings provide a neurobiological rationale for considering the DLPFC, Broca area, and adjacent cortical regions as key targets for NIBS to modulate cognition and language.

### 3.4 Networks related to emotion, pain, and sleep

Children with cerebral palsy have substantially higher rates of anxiety, depression, chronic pain, and sleep disturbances than typically developing children (38, 39). Although the underlying neurobiological mechanisms remain incompletely understood, accumulating evidence suggests that imbalance within prefrontal and limbic systems plays an important role (40, 41). In adults with depressive and anxiety disorders, abnormal functional connectivity between the dorsolateral prefrontal cortex (DLPFC) and regions such as the anterior cingulate cortex and amygdala is regarded as a core mechanism of impaired emotion regulation. Repetitive transcranial magnetic stimulation and tDCS targeting the DLPFC have been shown to improve mood and certain cognitive biases in these populations (42). Although direct evidence in children with cerebral palsy is limited, findings from chronic pain, neuropathic pain, and sleep disorder research in other populations suggest that persistent pain and sleep related symptoms may involve maladaptive cortical plasticity, altered thalamocortical dynamics, and disrupted sleep wake regulatory circuits (43). Moreover, evidence from other populations indicates that tDCS can alleviate chronic pain and insomnia by modulating activity in M1, S1, and the DLPFC, providing a theoretical basis for future investigation of NIBS as an adjunctive approach for comorbidity management in cerebral palsy (44, 45). The multisystem brain network alterations and candidate NIBS targets discussed above are summarized in Figure 1.

**Figure 1:**
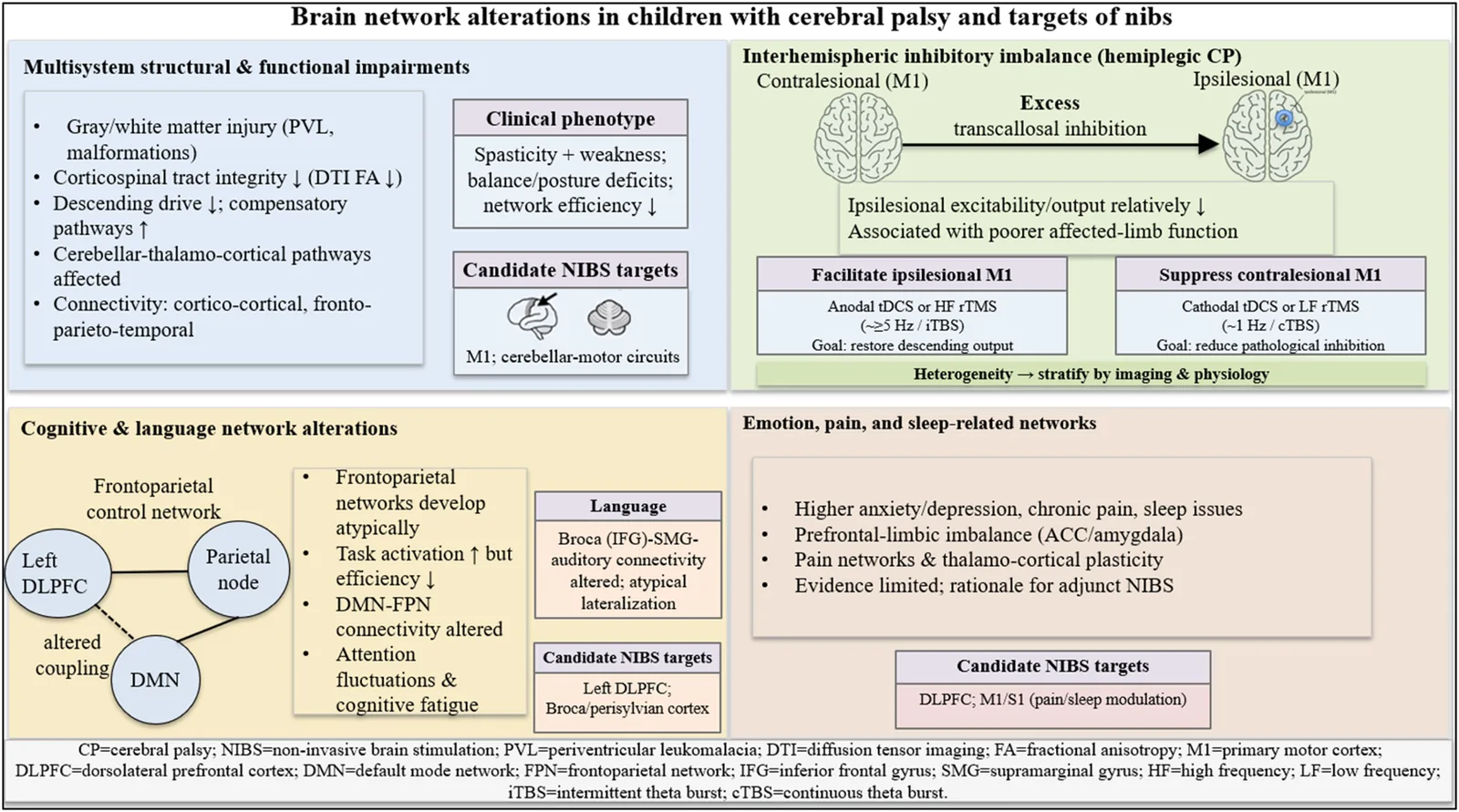
Brain network alterations in children with cerebral palsy and candidate targets for non-invasive brain stimulation.

## 4 Neurophysiological mechanisms of tdcs and rtms in children with cerebral palsy

### 4.1 Modulation of cortical excitability and synaptic plasticity

A shared feature of tDCS and rTMS is the ability to modulate excitability of targeted cortical regions under non invasive conditions and to induce durable changes in synaptic and network plasticity. tDCS applies a weak continuous current that can slightly depolarize or hyperpolarize neuronal membrane potentials, thereby altering the probability of neuronal firing during stimulation. Post stimulation effects can persist for tens of minutes to several hours and are commonly linked to NMDA receptor dependent mechanisms resembling long term potentiation and long term depression (46, 47).

Repetitive transcranial magnetic stimulation induces activity dependent changes in synaptic strength by delivering repetitive magnetic pulses that influence cortical excitability and inhibition. Stimulation frequency and pattern are key parameters that shape the direction of rTMS related plasticity. In general, low frequency protocols tend to reduce excitability, whereas higher frequency protocols tend to increase excitability, although responses vary across individuals and clinical phenotypes (48–50).

Moreover, the effects of rTMS are not restricted to the cortical area directly beneath the coil. They can propagate along structural and functional connections and reshape activity patterns at the network level (51). Studies combining TMS with functional MRI or EEG show that stimulation targeting M1 or prefrontal regions can alter connectivity and synchronization within motor and cognitive networks, and these network changes may relate to behavioral outcomes (52, 53). In practical terms, this supports choosing stimulation targets based on the functional network that needs support, rather than assuming a purely local effect. It also supports pairing stimulation with task specific training, because NIBS is more likely to facilitate learning during practice than to replace practice.

### 4.2 Interhemispheric inhibition model and the strategy of facilitating the ipsilesional hemisphere and suppressing the contralesional hemisphere

In hemiplegic cerebral palsy and in children with hemiparesis following perinatal stroke, imbalance in transcallosal interactions is relatively common. This imbalance is characterized by increased inhibition from the contralesional M1 to the ipsilesional M1, together with relatively reduced inhibition from the ipsilesional to the contralesional hemisphere. Such asymmetric inhibition is considered to constrain reorganization and motor output of the injured hemisphere (54, 55).

In cerebral palsy, emerging evidence supports the feasibility of approaches that facilitate the ipsilesional hemisphere or suppress excessive contralesional activity in combination with intensive upper limb training. Trials have reported improvements in fine motor outcomes when inhibitory low frequency rTMS over contralesional M1 is combined with constraint induced movement therapy, including gains in standardized upper limb measures and related functional tasks (56, 57). Systematic reviews and network meta analyses suggest an overall favorable signal for M1 targeted rTMS, although heterogeneity in stimulation parameters and study quality limits the certainty of conclusions (58, 59). Clinically, this model provides a rationale for selecting M1 based protocols in unilateral presentations, but it also highlights the need for stratification using neuroimaging or neurophysiology, because reorganization patterns differ across children and may influence responsiveness.

### 4.3 Modulation of frontoparietal networks and cognitive and dual task performance

The dorsolateral prefrontal cortex (DLPFC) and its connections with parietal regions form a core network for executive function, attentional allocation, and cognitive motor dual task control (60). Connectivity within this network is associated with working memory, attentional control, and dual task performance (61). Adult trials and meta analyses suggest that DLPFC targeted stimulation can improve executive outcomes and may benefit dual task gait, potentially through more efficient network activation and connectivity changes (62). These mechanistic links are largely derived from adult evidence and should be interpreted cautiously when applied to pediatric cerebral palsy, given developmental differences in network maturation and lesion related reorganization.

In children with cerebral palsy, a trial applying 10 Hz rTMS over the left DLPFC reported improved manual and cognitive performance under dual task conditions, with no serious adverse events (63). Evidence for tDCS remains limited. A pilot trial combining anodal DLPFC tDCS with cognitive training suggested feasibility and possible gains in cognition and language related measures, but the small sample limited between group conclusions (64). From a clinical perspective, DLPFC targeting is most relevant when the rehabilitation goal involves attention, executive control, and performance under real world cognitive load, rather than isolated strength or range of motion. Future studies should evaluate whether gains translate to daily participation outcomes such as classroom tasks, complex self care, and communication demands. The proposed neurophysiological mechanisms of tDCS and rTMS, including their effects on cortical excitability, interhemispheric inhibition, and network-level function, are summarized in Figure 2.

**Figure 2:**
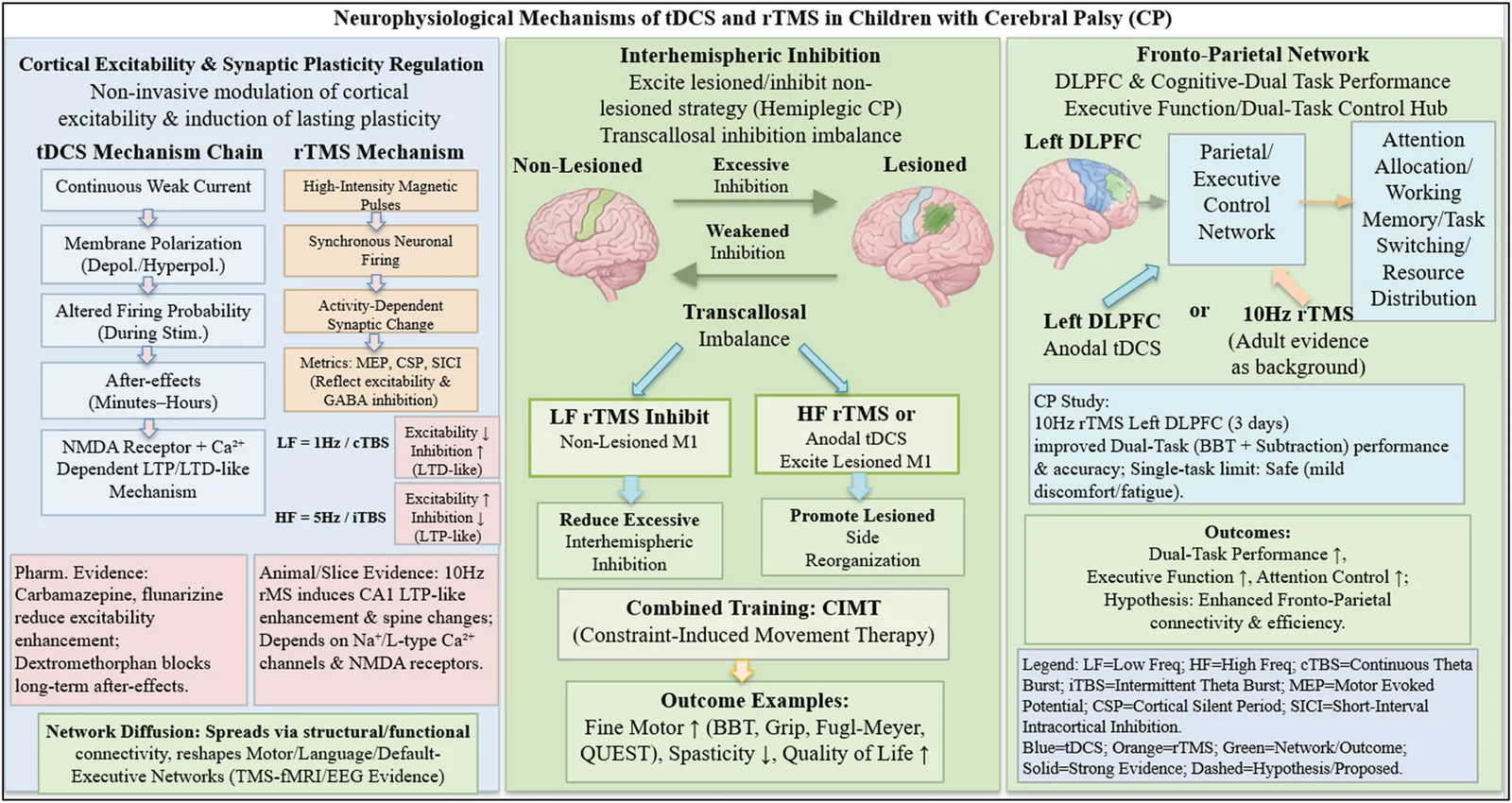
Neurophysiological mechanisms of tDCS and rTMS in cerebral palsy.

## 5 Evidence for clinical applications of noninvasive brain stimulation in children with cerebral palsy

Key systematic reviews and meta-analyses of non-invasive brain stimulation in children with cerebral palsy are summarized in Table 1.

**Table 1:** Structured summary of key reviews and meta-analyses on non invasive brain stimulation in children with cerebral palsy.

Type	Modality	Sample	GMFCS or MACS	Target	Stimulation parameters	Adjunct training and comparator	Primary outcomes	Key findings
Zhong et al. 2025 (review of RCTs with meta analysis) (16)	tDCS	tDCS trials: 11 RCTs, *n* = 260 (total review: 16 RCTs, *n* = 346)	GMFCS I 43.4 percent; II 40.8 percent; III 15.8 percent	Mainly M1 and gait related motor areas	Commonly 1 mA, 20 min per session, 10 sessions over 2 weeks; some 15 sessions	tDCS plus treadmill or gait training, physiotherapy, or VR balance training; comparator sham plus training or training alone	6 min walk test; 10 meter walk test; gait speed; step length; GMFM; Pediatric Balance Scale	Small to moderate improvements in mobility and gait; balance effects less consistent; adverse events mainly mild transient discomfort
Zhong et al. 2025 (review of RCTs with meta analysis) (16)	rTMS	rTMS trials: 5 RCTs, *n* = 86 (total review: 16 RCTs, *n* = 346)	GMFCS I 43.4 percent; II 40.8 percent; III 15.8 percent	Motor cortex and motor related regions	Protocols varied across studies	rTMS plus gait or balance training or usual rehabilitation; comparator sham or rehabilitation alone	GMFM; gait speed and related parameters; muscle tone measures	Positive trend for gross motor and gait outcomes, but limited trials and heterogeneity; safety generally acceptable
Sun et al. 2023 (systematic review and meta analysis) (69)	rTMS	29 trials, *n* = 988	GMFCS not consistently reported	Predominantly motor cortex targets; often contralesional or bilateral motor related regions	Most commonly 1 Hz or 5 Hz; course length and total dose varied	rTMS plus conventional rehabilitation or exercise training; comparator sham rTMS or usual rehabilitation	GMFM 88; fine motor outcomes; language response rate	Improvements reported for GMFM 88 and fine motor outcomes; pooled language response rate increased; substantial heterogeneity and variable study quality
Zhang et al. 2025 (systematic review and meta analysis) (17)	tDCS	15 studies included, 10 in meta analysis; total *n* = 366	MACS I to IV predominated; GMFCS not summarized	Most commonly contralesional non affected M1; some ipsilesional more affected M1	Typically 0.7 to 1.5 mA, 20 min per session, 2 to 10 sessions per course	tDCS plus CIMT, PT, OT, or task oriented upper limb training; comparator sham or training alone	Assisting Hand Assessment; Box and Block Test; Canadian Occupational Performance Measure; Modified Ashworth Scale; ROM; grip strength	Safety and tolerability generally good; signals for improved manual dexterity and reduced spasticity in some outcomes; heterogeneity limits confidence
Zhang et al. 2025 (systematic review and meta analysis) (17)	rTMS	15 studies included, 10 in meta analysis; total *n* = 366	MACS I to IV predominated; GMFCS not summarized	Most commonly contralesional non affected M1; some ipsilesional more affected M1	Example protocol: 90 percent resting motor threshold, 1 Hz, 1200 pulses, 10 sessions over 2 weeks; protocols varied	rTMS plus CIMT, PT, OT, or task oriented upper limb training; comparator sham or training alone	Assisting Hand Assessment; Box and Block Test; Canadian Occupational Performance Measure; Modified Ashworth Scale; ROM; grip strength	Safety and tolerability generally good; effects uncertain due to limited trials and heterogeneity
Fernández Pérez et al. 2025 (systematic review of RCTs) (76)	tDCS	12 RCTs, *n* = 291 (CP 281); included a small number of pediatric stroke participants	MACS reported in 236 participants: I 24.4 percent; II 47.8 percent; III 6.5 percent; IV 2.4 percent	tDCS montages varied, including M1 to supraorbital and bilateral M1	1 to 15 sessions; substantial protocol heterogeneity	Delivered alone or combined with upper limb rehabilitation such as CIMT; comparator sham or rehabilitation alone	Upper limb motor activity outcomes	Stimulation alone did not reliably improve upper limb motor activity; combined protocols with intensive training showed more favorable signals; evidence heterogeneous
Fernández Pérez et al. 2025 (systematic review of RCTs) (76)	rTMS	12 RCTs, *n* = 291 (CP 281); included a small number of pediatric stroke participants	MACS reported in 236 participants: I 24.4 percent; II 47.8 percent; III 6.5 percent; IV 2.4 percent	rTMS mainly targeted contralesional or non affected M1	Commonly 1200 pulses per session, 5 to 10 sessions; protocol heterogeneity	Delivered alone or combined with upper limb rehabilitation such as CIMT; comparator sham or rehabilitation alone	Upper limb motor activity outcomes	Stimulation alone did not reliably improve upper limb motor activity; combined protocols with intensive training showed more favorable signals; evidence heterogeneous

tDCS, transcranial direct current stimulation; rTMS, repetitive transcranial magnetic stimulation; M1, primary motor cortex; DLPFC, dorsolateral prefrontal cortex; GMFM, Gross Motor Function Measure; GMFCS, Gross Motor Function Classification System; MACS, Manual Ability Classification System; CIMT, constraint induced movement therapy; PT, physical therapy; OT, occupational therapy; VR, virtual reality; ROM, range of motion.

### 5.1 Lower limb function, gait, and balance

Most lower limb studies applied anodal tDCS over primary M1 combined with gait or balance training. In a double blind randomized controlled trial, Duarte and colleagues randomized 24 children with spastic cerebral palsy aged 5 to 12 years to anodal tDCS over lesioned side M1 plus treadmill training or sham plus treadmill training for 10 sessions over 2 weeks. Functional severity may influence responsiveness, and GMFCS levels should be considered when interpreting outcomes. The trial stratified randomization by GMFCS levels I to II versus level III (65). The active group showed greater improvements in Pediatric Balance Scale and postural sway measures, with partial maintenance at 1 month follow up. Related trials using 1 to 2 mA for 20 min per session combined with treadmill or gait training reported improvements in gait speed, step length, and 6 min walking distance, with favorable safety profiles (66). A single session study combining anodal M1 tDCS with virtual reality based gait and balance training reported immediate improvements in selected postural sway parameters (67).

Across trials, systematic reviews and meta analyses suggest that anodal M1 tDCS combined with lower limb training yields small to moderate improvements in gait and gross motor function, while effects on balance are less consistent (16, 68). Lower limb rTMS studies typically use low frequency stimulation to suppress contralesional M1 or high frequency stimulation to facilitate ipsilesional M1, combined with conventional therapy. Small trials and pooled analyses indicate potential benefits for gross motor function and muscle tone, but evidence is limited by small samples and variable parameters, so rTMS remains a promising adjunct requiring further optimization (69).

### 5.2 Upper limb function and manual dexterity

Upper limb studies commonly combine CIMT or structured upper limb training with tDCS or rTMS targeting M1. In a sham controlled trial, Gillick and colleagues applied cathodal tDCS over nonlesioned M1 plus CIMT versus sham plus CIMT. Both groups improved, while the tDCS group showed larger effect sizes in selected fine motor and bimanual coordination tasks, with neurophysiological signals consistent with increased corticospinal excitability in the lesioned hemisphere (70).

For rTMS, Wu and colleagues applied inhibitory low frequency rTMS over contralesional M1 combined with modified CIMT in preschool children with unilateral cerebral palsy. Improvements were reported in Assisting Hand Assessment and related measures, with no serious adverse events (56). A systematic review concluded that combining tDCS or rTMS with upper limb training is generally safe and feasible and may produce moderate effects, but overall evidence quality is low to moderate due to small samples and protocol heterogeneity, supporting the need for larger multicenter trials (69).

### 5.3 Modulation of spasticity and abnormal muscle tone

Spasticity outcomes are often secondary endpoints in NIBS studies. Systematic reviews suggest that tDCS, commonly anodal M1 or cerebellar stimulation combined with physical therapy, may reduce Modified Ashworth Scale scores in the short term and may improve muscle stiffness and joint range of motion, but heterogeneity limits conclusions about dose response (17, 58). Network meta analysis findings suggest that low frequency rTMS over contralesional M1 may be more favorable for reducing muscle tone and improving hand function, whereas high frequency rTMS over ipsilesional M1 may be more favorable for gross motor recovery (59).

#### 5.3.1 Cognitive function and dual task capacity

Recent studies increasingly target DLPFC and frontoparietal executive control networks to address attention, executive function, and cognitive motor dual task performance. Adult evidence suggests that anodal DLPFC tDCS or excitatory rTMS can improve working memory, attentional control, and dual task gait performance, with changes linked to frontoparietal connectivity and activation efficiency, providing a rationale for pediatric investigation (71, 72).

In children with cerebral palsy, Ko et al. conducted a pilot sham controlled trial in 13 children with comorbid cognitive impairment. Anodal DLPFC tDCS plus cognitive training was feasible and well tolerated and was associated with within group improvements in cognition, activities of daily living, and language related measures, although between group differences were not significant (64). Evidence for pediatric dual task outcomes remains limited. One randomized trial combined anodal motor cortex tDCS with cognitive motor dual task training in 30 children and reported greater improvements than sham in functional activity capacity, selected cognitive measures, and cortical excitability, with persistence at 1 month follow up and no serious adverse events (73). Overall, DLPFC and executive network targeted NIBS combined with structured cognitive or dual task training shows acceptable feasibility and signals of benefit, but evidence remains exploratory due to small samples and heterogeneous protocols. Larger studies with mechanistic endpoints and participation relevant outcomes are needed before firm clinical recommendations can be made.

#### 5.3.2 Language and communication outcomes

Language and communication impairments contribute substantially to participation restrictions in children with cerebral palsy, but evidence for NIBS in this domain is limited. Available studies suggest feasibility and acceptable safety when stimulation is combined with language or communication oriented training, with preliminary efficacy signals. In children with cerebral palsy and cognitive impairment, anodal DLPFC tDCS plus cognitive training was associated with improvements in language related measures, suggesting indirect facilitation of language processing (64). A small prospective study reported that left DLPFC tDCS combined with augmentative and alternative communication training improved Communication Matrix levels and spontaneous expressive behaviors, with generally mild transient adverse effects (74). Evidence from broader pediatric developmental language delay also suggests added benefits of tDCS combined with language and cognitive training, supporting cautious extrapolation to cerebral palsy (75). For rTMS, meta analytic evidence suggests potential improvement in language response rates compared with speech therapy alone, but prescriptions vary and overall evidence quality remains limited (69).

#### 5.3.4 Phenotype coverage, dystonia, and other movement disorder presentations

The current evidence base for NIBS in children with cerebral palsy is unevenly distributed across clinical phenotypes. Most available therapeutic studies have enrolled children with spastic CP, particularly those with unilateral involvement, and ambulatory participants, commonly within GMFCS levels I to III. Studies targeting lower limb function, gait, and balance have also included children with spastic diplegic or bilateral spastic CP. Therefore, the existing evidence is not restricted to unilateral CP alone, but the strongest and most consistently reported findings are derived from spastic and relatively ambulatory populations. In contrast, evidence for dyskinetic, ataxic, mixed, and more severe non-ambulatory CP remains limited (17, 76).

Motor dysfunction in unilateral CP is often discussed in relation to corticospinal tract injury and interhemispheric inhibitory imbalance, providing a rationale for M1-based stimulation strategies (28, 29). However, this model may not adequately explain other CP phenotypes. Dyskinetic CP is characterized by fluctuating muscle tone, involuntary movements, and impaired motor control, and is commonly associated with abnormalities involving the basal ganglia, thalamocortical pathways, and broader sensorimotor networks (77, 78). TMS has been used to investigate corticospinal excitability, inhibitory circuitry, and motor network physiology in CP and other pediatric movement disorders. In addition, rTMS and tDCS have been explored as non-invasive neuromodulation approaches for dystonia in broader clinical populations, although the reported effects are modest and protocol dependent (79). These pathophysiological differences suggest that evidence derived from M1-centred protocols in unilateral or spastic CP should not be directly generalized to dyskinetic CP or other movement disorder presentations. For dyskinetic, dystonic, ataxic, mixed, and more severe non ambulatory presentations, the available evidence remains preliminary, and clinical applicability should be considered uncertain.

#### 5.3.5 Emerging complementary technology assisted rehabilitation strategies

Although this review focuses primarily on tDCS and rTMS, several non invasive and technology assisted rehabilitation approaches are increasingly being used to enhance motor learning, sensory feedback, engagement, and task specific practice in children with cerebral palsy. Mechanical and vibration based interventions, including whole body vibration and local vibrotactile stimulation, may influence proprioceptive input, muscle activation, postural control, and sensorimotor integration (80, 81). Game based and virtual reality assisted rehabilitation provide enriched, repetitive, and feedback driven environments that may increase motivation and support motor learning. Systematic reviews and meta analyses indicate that VR assisted or exergame based interventions may improve selected outcomes related to balance, upper and lower limb function, daily activity performance, and cognition, although effects differ according to immersion level, task design, training dose, and comparator intervention (82, 83). Other technology assisted approaches, including robotic gait or upper limb training, functional electrical stimulation or neuromuscular electrical stimulation, wearable haptic feedback systems, and brain computer interface based rehabilitation, may also contribute to intensive and individualized training by providing assistive movement, sensory feedback, task repetition, or closed loop control (84, 85).

## 6 Limitations and future directions

This review has several limitations. Although it was informed by a structured literature search, it was designed as a narrative review rather than a systematic review. Therefore, no formal risk of bias assessment, certainty of evidence grading, or quantitative meta analysis was conducted. In addition, the available studies varied substantially in participant characteristics, CP subtype, lesion profile, stimulation modality, target selection, stimulation parameters, adjunct rehabilitation protocols, outcome measures, and follow up duration, which limits objective comparison across studies. Some mechanistic interpretations were also supported by evidence from adult or non CP populations when pediatric CP specific evidence was limited. Accordingly, the findings should be interpreted as a structured narrative synthesis of current evidence rather than definitive evidence for clinical implementation.

Future research should move beyond feasibility studies toward mechanism informed and phenotype stratified trials that improve reproducibility and clinical translation. Given the heterogeneity of lesion type, developmental stage, motor severity, and network reorganization, neuroimaging and neurophysiological markers may help guide target selection and protocol matching. Candidate markers include DTI measures of corticospinal and cerebello thalamo cortical integrity, functional connectivity within motor and frontoparietal networks assessed by functional MRI or functional near infrared spectroscopy, and TMS measures of interhemispheric inhibition and cortical excitability. Large multicenter randomized controlled trials are needed to standardize stimulation parameters, model dose response, and clarify the optimal timing of stimulation relative to task based training. Outcome assessment should extend beyond impairment scales to include participation, real world dual task performance, quality of life, caregiver burden, longer term maintenance, and pediatric safety monitoring during repeated NIBS applications.

## 7 Conclusion

Overall, this narrative review indicates that tDCS and repetitive transcranial magnetic stimulation are generally safe and well tolerated in children with cerebral palsy when applied within recommended parameter ranges. When combined with intensive rehabilitation training, both approaches show small to moderate positive signals for outcomes including gross motor function, gait, selected balance measures, and upper limb manual function. Targeted stimulation based on frontoparietal control and language networks also appears feasible and suggests a potential augmentation trend for cognition, dual task performance, and communication outcomes. However, current studies are commonly limited by small sample sizes, substantial heterogeneity in stimulation parameters and training protocols, lack of unified standards for target localization and treatment course design, short follow up periods, and insufficient assessment of real world participation outcomes. As a result, the current evidence base remains insufficient to support broad clinical recommendations. Future work incorporating mechanism and phenotype driven stratification, high quality multicenter randomized controlled trials, parameter standardization, and long term validation of safety and efficacy is expected to clarify optimal stimulation strategies and boundaries of benefit across cerebral palsy subtypes, and to provide a stronger evidence foundation for augmenting rehabilitation of higher order functions, communication, and participation in complex real world contexts.
